# Second window ICG predicts postoperative MRI gadolinium enhancement in high grade gliomas and brain metastases

**DOI:** 10.3171/2021.10.FOCVID21204

**Published:** 2022-01-01

**Authors:** Ritesh Karsalia, Nina H. Cheng, Clare W. Teng, Steve S. Cho, Stefan Harmsen, John Y. K. Lee

**Affiliations:** 1 Department of Neurosurgery, Hospital of the University of Pennsylvania, Philadelphia;; 2 Perelman School of Medicine at the University of Pennsylvania, Philadelphia;; 3 Drexel University College of Medicine, Philadelphia, Pennsylvania; and; 4 Department of Neurosurgery, Barrow Neurological Institute, Phoenix, Arizona

**Keywords:** indocyanine green, fluorescence-guided surgery, brain tumor, NIR-II window, NIR fluorescence

## Abstract

A prospective trial evaluating the utility of second window indocyanine green (SWIG) in predicting postoperative MRI gadolinium enhancement was performed on high-grade gliomas (HGGs) and brain metastases. Compared to white light alone, SWIG demonstrated a higher sensitivity, negative predictive value, and accuracy in predicting residual neoplasm on MRI. The specificity of SWIG for predicting MRI enhancement was higher in HGGs than brain metastases. Clinically, near-infrared (NIR) imaging was better able to predict tumor recurrence than postoperative MRI. These results illustrate how SWIG is able to take advantage of gadolinium-like distribution properties to extravasate into the tumor microenvironment, enabling guidance in surgical resection.

The video can be found here: https://stream.cadmore.media/r10.3171/2021.10.FOCVID21204

## Transcript

In this video presentation, we discuss the application of second window indocyanine green (SWIG) in predicting postoperative MRI gadolinium (Gd) enhancement in both high-grade gliomas (HGGs) and brain metastases. What makes indocyanine green (ICG) unique in the context of intraoperative visualization is that it is a near-infrared (NIR) fluorophore, which confers ICG with some unique benefits in the surgical setting. ICG is excited around 805 nm, and its emission is detected around 835 nm. ICG is thought to accumulate due to the enhanced permeability and retention effect in the tumor microenvironment, due to angiogenesis and the disruption of blood-brain barrier integrity, and it may be endocytosed directly by the tumor cells.^1,2^ The supposed mechanism of ICG distribution closely mimics that of gadolinium in contrast-enhanced imaging studies, potentially allowing us to utilize its NIR fluorescence as a proxy for postoperative MRI enhancement.^1–6^ This property would allow for the use of ICG as an adjunct or alternative to current neuronavigation and intraoperative MRI techniques, which are not always reliable or easily accessible.^7^

### 1:30 Methods.

Our study took place between 2014 and 2019 and included over 350 patients, with a variety of CNS tumors. The technique we used is named “second window ICG” because we administered the dye in high doses (up to 5 mg/kg) up to 24 hours before surgery. Intraoperative NIR imaging was performed using an exoscope, and the residual fluorescence postresection was compared to Gd enhancement on postoperative MRI. Importantly, NIR imaging did not guide the extent of surgical resection actually performed by the surgeon.

### 2:04 Second Window ICG.

ICG is more traditionally used in the context of video angiography in typical doses between 0.2 and 0.5 mg/kg, with imaging performed within minutes of administration. Our technique and application of ICG is termed “second window ICG” because we administer high doses of ICG (between 2.5 and 5 mg/kg) 24 hours before surgery. We are able to take advantage of the enhanced permeability and retention effect in the tumor microenvironment to detect the fluorescence of ICG in this second window, as seen on the diagram.

### 2:40 Results: High-Grade Gliomas.

For the high-grade glioma group, we had 34 patients with NIR imaging of the final surgical cavity view postresection. Of the 25 patients who had positive NIR signal in the final view, 23 of these patients had correlating residual neoplasm on postoperative MRI. Of the 9 patients with no residual NIR fluorescence in the final view, 8 had a complete resection of enhancing tumor on postoperative MRI. When compared to white light alone, SWIG ICG NIR imaging had a higher sensitivity (96%), negative predictive value (89%), and accuracy (91%) in predicting residual neoplasm on MRI.

### 3:20 Case History.

Here we have a case highlighting the utility of SWIG in predicting postoperative MRI. This patient was a 71-year-old-man presenting with progressive confusion over a 3-week period, having specific difficulties with speech. His primary care provider thought he had a stroke and referred the patient for an MRI.

### 3:38 Preoperative MRI.

Preoperative imaging reveals a 38-mm necrotic left parietal mass in the left parietal lobe, presumably glioblastoma. Here’s a still of the contrast-enhanced MRI further showing the tumor. The patient was started on dexamethasone for three times per day.

### 3:56 SWIG-Guided Surgery in Left Parietal Craniotomy.

Here we are performing the craniotomy for this patient. The fluorescence seen in this video is a pseudocolor overlay on top of white light. One of the unique advantages of ICG is its ability to penetrate through the dura mater, given that it has a longer wavelength than visible light dyes.^4,6^ This transdural fluorescence allows us to center over the tumor prior to incising the dura. Once the dura has been opened, we can see the dye permeating the hypervascular tumor environment, allowing the surgeon to identify the neoplastic tissue.

### 4:55 Final View ICG Demonstrating Residual Fluorescence.

Once the majority of the tumor has been removed, we can see some residual fluorescence in this final view. As previously mentioned, fluorescence was not used to guide the extent of resection in the study. Here, the surgeon removes a sample, which is clearly visualized by ICG, from the margin of the cavity to send to pathology for further evaluation.

### 5:31 Postoperative MRI Illustrating Residual Enhancement.

Postoperative MRI shows some residual enhancement around the resection cavity, deep and close to the lateral ventricle. These enhancements are highlighted by the white arrows.

### 5:45 Correlation Between Final View NIR Fluorescence and Gd Enhancement.

This is a comparison highlighting the correlation between SWIG fluorescence and Gd enhancement. Prior to the resection, on the left, we can see the fluorescence in the bulk of the tumor correlating to the enhancement on MRI. Postresection, on the right, we can see the location of residual fluorescence (near the lateral ventricle) correlating to the rim of enhancement also seen on postoperative MRI. Ultimately, this patient underwent combined chemoradiation following surgery due to this residual tumor.

### 6:17 Results: Brain Metastases Group.

For the brain metastases group, we had 44 patients with NIR imaging of the final surgical cavity postresection. This group highlights that a lack of NIR fluorescence in the final view correlates with a lack of Gd enhancement on postoperative MRI, emphasizing the sensitivity and negative predictive value seen with SWIG. Of the 16 patients in our study with no residual ICG fluorescence upon resection, all 16 had a complete resection of enhancing tumor on postoperative MRI. Though compared to the HGG groups, SWIG has a lower specificity for predicting MRI enhancement in brain metastases, NIR imaging clinically predicted the tumor recurrence better than postoperative MRI. Of the patients with recurrent tumors, a greater number had positive findings on final view NIR imaging compared to postoperative MRI.

### 7:12 Final View NIR Fluorescence and Gd Enhancement in GTR and non-GTR.

This figure shows the correlation between final view ICG and postoperative Gd enhancement in a gross-total resection (GTR) and non–gross-total resection (non-GTR).^8^ The top row depicts a metastasis in the right temporal lobe. Upon resection, there is no visible NIR fluorescence in the surgical cavity (seen in box E on the top right). This correlates with the lack of significant enhancement seen in the postoperative MRI (box B).^8^ In contrast, the bottom row shows a non-GTR for a right occipital mass. Box J (on the bottom right) highlights residual enhancement seen anteriorly in the resection cavity in the final view, which correlates to the anteriorly located enhancement seen in the postoperative MRI.^8^

### 8:00 Conclusions.

In conclusion, SWIG extravasates into the tumor microenvironment of HGGs and brain metastases via the enhanced permeability and retention effect, with gadolinium-like distribution properties. SWIG provides an intraoperatively useful means of predicting postoperative Gd enhancement with a high sensitivity and NPV.^6^ In this context, SWIG serves as a valuable tool to improve overall sensitivity and achieve a greater extent of resection, which may have positive implications related to survival and adjuvant therapy.^5,9^

## Disclosures

Dr. Lee reported other from VisionSense/Medtronic during the conduct of the study.

## Author Contributions

Primary surgeon: Lee. Editing and drafting the video and abstract: Karsalia, Cheng, Cho, Harmsen. Critically revising the work: Karsalia, Cheng, Cho, Harmsen. Reviewed submitted version of the work: Karsalia, Cheng, Teng, Harmsen. Supervision: Karsalia, Teng.
